# Insulin resistance may accelerate typical changes in heart function among type 1 diabetes patients, particularly in overweight patients: a preliminary study

**DOI:** 10.3389/fendo.2024.1384514

**Published:** 2024-05-21

**Authors:** Klaudia Czarnik, Zbigniew Sablik, Anna Borkowska, Jarosław Drożdż, Katarzyna Cypryk

**Affiliations:** ^1^ Department of Internal Diseases and Diabetology, Medical University of Lodz, Lodz, Poland; ^2^ II Department of Cardiology, Medical University of Lodz, Lodz, Poland; ^3^ Department of Digestive Tract Diseases, Faculty of Medicine, Medical University of Lodz, Lodz, Poland

**Keywords:** diabetes type 1, diabetes complications, diastolic heart failure, insulin resistance, lipid accumulation product

## Abstract

**Introduction:**

Type 1 diabetes (T1D) is a metabolic disease characterized by insulin deficiency and subsequent hyperglycemia. Cardiovascular diseases are the prime cause of mortality and morbidity among patients with T1D. Accumulating metabolic disturbances and accelerated cardiac fibrosis fuel the development of heart dysfunction. As insulin resistance (IR) is a risk factor for the development and worsened course of heart failure, this study aimed to assess its impact on heart function in patients with T1D.

**Methods:**

Adult participants were recruited prospectively. The inclusion criteria included a diagnosis of T1D. The exclusion criteria were other types of diabetes, symptoms/treatment of heart failure, AST and/or ALT exceeding the upper reference limit by ≥2x, hepatitis, alcoholism, metformin treatment, and pregnancy. The participants underwent a medical interview, physical examination, biochemical test, and echocardiography.

**Results:**

The mean age in the study group was 38 ± 9.6 years, and the mean diabetes duration was 21.8 ± 11.3 years. The median BMI in the study cohort was 23.39 kg/m^2^. Patients with IR had significantly lower mitral E/A ratio and left ventricular and left atrial volume ratio (LVLAVR), higher LV mass index, and presented with altered mitral annular velocities.

**Conclusions:**

IR seems to accelerate the pattern of typical changes in heart function among patients with T1D, especially in the overweight subgroup.

## 1 Introduction

Type 1 diabetes (T1D) is a metabolic disease characterized by insulin deficiency following the autoimmune destruction of pancreatic β-cells, resulting in hyperglycemia (1). Its prevalence seems to rise and varies regionally from 0.6 cases per 1,000 people in Asia to 3.0 per 1,000 in Europe and to 4.4 per 1,000 in North America (2). In the cohort of patients with T1D, cardiovascular diseases are considerably the leading cause of mortality and morbidity (3).

As a consequence of the complex interactions between metabolic disturbances such as insulin resistance, increased oxidative stress, cardiomyocyte low-grade inflammation, and disturbed signaling, patients with T1D are prone to developing specific cardiac dysfunction called *diabetic cardiomyopathy* early in the course of diabetes and in the absence of other risk factors such as hypertension (4, 5). No previously tested method, applicable to large cohorts, such as measuring B-type natriuretic peptide, was considered sufficiently sensitive and specific in discriminating patients with early stages of diabetic cardiomyopathy. Hence, there are no screening protocols to identify such patients (6).

Patients in the early stages of diabetic cardiomyopathy usually present with diastolic heart failure, which may be characterized by a markedly altered early diastolic mitral inflow velocity to late diastolic mitral inflow velocity ratio (mitral E/A ratio) and increased early diastolic mitral inflow velocity to mean mitral annulus early diastolic velocity ratio (E/e′ ratio) (7).

Insulin resistance (IR) is an established risk factor for the development and worsened course of heart failure (8). Given the growing number of observed rates of so-called double diabetes, which describes the patients with T1D concomitantly presenting with features of insulin resistance traditionally linked to type 2 diabetes (T2D), this study aimed to preliminarily investigate the correlation between IR and heart dysfunction in a cohort of patients with T1D without symptoms of heart failure (HF) (9–11).

## 2 Methods

The study participants were recruited prospectively and consecutively at the Diabetology Clinic over the period from October 1, 2021 to September 1, 2022. The inclusion criteria were diagnosis of type 1 diabetes and age of 18 years or older. The exclusion criteria consisted of diagnosis of any other type of diabetes, symptoms of heart failure, treatment of heart failure, active hepatitis, alcoholism, metformin treatment, pregnancy, and aspartate aminotransferase (AST) or alanine aminotransferase (ALT) concentrations, or both, of at least two times the upper reference limit.

After obtaining informed consent, medical data and anthropometric measurements including body weight, height, and waist circumference were collected. Body mass index (BMI) was calculated as body weight expressed in kilograms divided by height in meters squared (12).

### 2.1 Biochemistry

In order to establish the patients’ present health status, full blood, plasma, serum, and urine tests examining metabolic parameters, cardiac, liver, and thyroid function, as well as diabetic complications, including chronic kidney disease, were performed after an 8-h fast. Concentrations of glycated hemoglobin and C-reactive protein (CRP) were determined using an immunoturbidimetric assay (DxC, Beckman Coulter). Thyroid-stimulating hormone (TSH) level was measured using the chemiluminescent immunoassay method (Alinity, Abbott). Aspartate aminotransferase (AST), alanine aminotransferase (ALT), gamma-glutamyltransferase (GTP), creatinine, lipids, and uric acid quantities were determined using the spectrophotometric method (DxC AU, Beckman Coulter). The N-terminal prohormone of brain natriuretic peptide (NT-proBNP) level was assessed using the chemiluminescent immunoassay method (Cobas, Roche). The albumin/creatinine ratio (ACR) was determined using immunoturbidimetric and isotope dilution mass spectrometry methods (AU, Beckman Coulter). Finally, growth differentiation factor 15 (GDF-15) and apolipoprotein C3 (Apo-CIII) concentrations were measured using Human ELISA Kits (Biorbyt Ltd., Cambridge, United Kingdom).

The fatty liver index (FLI), a surrogate marker of nonalcoholic fatty liver disease (NAFLD) risk, was calculated using the following formula: FLI = (e^0.953 × ln(TG concentration in mmol/L) + 0.139 × BMI + 0.718 + ln(GGT concentration in U/L) + 0.053 × waist circumference in cm – 15.745^)/(1 + e^0.953 × ln(TG concentration in mmol/L) + 0.139 × BMI + 0.718 + ln(GGT concentration in U/L) + 0.053 × waist circumference in cm – 15.745^) × 100 (13).

### 2.2 Echocardiography

All enrolled patients were examined in the same institution by one experienced physician (ZS) using a commercially available ultrasound machine (GE Vivid Q, General Electric). The obtained images were then digitally stored. The performed measurements were done according to recommendations by the European Association of Cardiovascular Imaging (14).

Left and right ventricles were measured using linear M-mode tracing. The chamber diameters and volumes were determined in a four-chamber view using two-dimensional imaging. The ejection fraction was calculated by utilizing the biplane method of disk summation technique. Left ventricle mass was calculated by applying the cube formula to linear measurements from M-mode. Early diastolic mitral inflow velocity (mitral E) and late diastolic mitral inflow velocity (mitral A) were measured using pulsed-wave Doppler in an apical four-chamber view. Tissue Doppler velocities [lateral and septal mitral annulus early diastolic velocity (e′), peak systolic velocity (s′), and peak late diastolic velocity (a′)] were also measured in apical four-chamber view. Tricuspid annular plane systolic excursion (TAPSE) was obtained using M-mode tracing from the apical approach. The deceleration time of mitral E velocity (DT of mitral E velocity) and isovolumic relaxation time (IVRT) were measured with pulsed-wave Doppler in the apical four-chamber view. Furthermore, the mitral E/A ratio and the mitral E/e′ ratio were calculated (15, 16).

The left ventricular (LV)–left atrial (LA) volume ratio (LVLAVR) was calculated as the ratio of left atrial end-diastolic volume to left ventricular end-diastolic volume (17).

### 2.3 Insulin resistance

Since lipid accumulation product (LAP) offers one of the best reliability in identifying insulin resistance among surrogate insulin sensitivity indexes using neither glucose nor insulin concentrations, we decided to use it to detect insulin resistance in our study cohort (18). The LAP was calculated for men as (waist circumference in centimeters – 65) × (TG concentration in mmol/L) and for women as (waist circumference in centimeters – 58) × (TG concentration in mmol/L) (19). The cutoff value for insulin resistance (IR) was set at LAP ≥42.5 (20). On this basis, we divided the study participants into insulin-resistant (IR) and non-insulin-resistant groups (non-IR).

### 2.4 Statistical analysis

Statistical analyses were performed using Statistica 13.1 software (StatSoft Inc., Tulsa, OK, USA). As no previous research precisely described the issue we aimed to explore, we were solely able to roughly calculate the number of study participants. Based on the data presented in the paper by Giuseppina Novo et al. regarding mitral E/A in groups considered insulin-resistant and non-insulin-resistant, we estimated the total number of participants needed to equal 44 (21).

To confirm the normal distribution of data, we used the Shapiro–Wilk test. Continuous variables were then compiled as the mean and standard deviation (SD) or median and interquartile interval (25th and 75th percentile) according to their distribution. Subsequently, the above-mentioned parameters were calculated for the insulin-resistant and non-insulin-resistant groups. Afterward, we compared normally distributed data using unpaired Student’s *t-*test and nonparametric data using the Mann–Whitney *U*-test. Additionally, to assure the consistency of the data, we performed the Mann–Whitney *U*-test to compare the BMI in subgroups with normal weight, overweight, and obese individuals. To explore correlations between continuous variables and the mitral E/A and the mitral E/e′, we used Spearman’s *r* correlations. Then, univariable and multivariable linear regression models were performed to analyze the clinical predictors of worsened E/A and LV/LA ratios using all manually selected predictors and parameters selected by applying the backward stepwise approach with *p* to enter and remove set to 0.05. Statistical significance was set at the *p* < 0.05 level.

## 3 Results

A total of 65 patients with T1D met the inclusion criteria and were invited to the study. There were 10 of the patients excluded due to missing data. The clinical characteristics of the 55 participants included in the study are shown in **Table 1**. The mean age in the study cohort was 38 years (SD 9.6 years), and the mean diabetes duration was 21.8 years (SD 11.3 years). The patients in our study, for the most part, were of normal weight, and the median BMI in the study cohort was 23.39 kg/m^2^ (IQR 21.61–27.25 kg/m^2^). On average, the study participants presented with normal albuminuria (median ACR 4.45 mg/g, IQR 2.55–12.55 mg/g), the liver function tests were within normal ranges [median AST 20.95 U/L (IQR 17.05–26.15 U/L), median ALT 18.10 U/L (IQR 13.25–27.00 U/L), GGTP 19.70 U/L (IQR 12.20–37.95 U/L)] and low CRP levels (median CRP 2.25 mg/L, IQR 0.9–4.55 mg/L).

**Table 1:** Clinical characteristics of the study group.

Parameter	Mean (SD)	Median (IQR)
Baseline characteristics		
Age (years)	38.0 (9.6)	
Height (cm)	168.9 (9.9)	
Body weight (kg)		67.0 (60.0–79.0)
Waist circumference (cm)		80 (76–90)
BMI (kg/m^2^)		23.39 (21.61–27.25)
Diabetes duration (years)	21.8 (11.3)	
DDI (U)		40 (32–56)
DDI per body weight kg (U/kg)		0.62 (0.46–0.73)
Biochemistry		
HbA1c (%)		8.05 (7.15–9.90)
ACR (mg/g)		4.45 (2.55–12.55)
AST (U/L)		20.95 (17.05–26.15)
ALT (U/L)		18.10 (13.25–27.00)
GGTP (U/L)		19.70 (12.20–37.95)
NT-proBNP (pg/mL)		46.05 (27.00–77.10)
CRP (mg/L)		2.25 (0.90–4.55)
uric acid (umol/L)		255.65 (200.90–325.20)
eGFR (mL/min/1.73 m^2^)	104.7 (19.9)	
Total cholesterol (mmol/L)	4.87 (0.98)	
LDL (mmol/L)	2.90 (0.74)	
HDL (mmol/L)	1.52 (0.34)	
Non-HDL (mmol/L)	3.34 (0.92)	
TG (mg/dL)		89.90 (66.43–124.88)
TSH (µIU/mL)		1.57 (0.83–2.20)
Insulin resistance		
LAP index		19.05 (10.40–38.00)
Fatty liver disease		
FLI		15.17 (6.84–43.07)
Experimental biomarkers		
GDF-15 (pg/mL)		291.97 (116.62–529.18)
APOC3 (ng/mL)		23.23 (15.80–33.43)
Echocardiography		
RVDD (mm)	25 (3)	
IVSD (mm)	13 (2)	
LVDD (mm)	46 (4)	
LVSD	30 (4)	
EF	65% (4%)	
LA vol (mL)	45.0 (11.4)	
LA vol index (mL/m^2^)	24.1 (4.8)	
Mitral E (cm/s)	81 (20)	
Mitral A (cm/s)	64 (17)	
Mitral E/A	1.25	1.04–1.67
Mitral DT (ms)	230	200–245
LV EDV (mL)	79 (15)	
LV ESV (mL)	28 (6)	
LV mass (g)	131.6 (35.4)	
LV mass index (g/m^2^)	71.1 (13.0)	
TDI bas sept e′ (cm/s)	11 (3)	
TDI bas sept s′ (cm/s)		8.5 (8–9)
TDI bas sept a′ (cm/s)		8 (7–10)
TDI bas lat e′ (cm/s)		14 (11–16)
TDI bas lat s′ (cm/s)		10 (9–12)
TDI bas lat a′ (cm/s)	8 (3)	
Mean e′ (cm/s)	12 (3)	
E/e’		6.75 (5.40–8.00)
RV TAM (mm)	26 (3)	
Vp (cm/s)	50 (6)	
LVLAVR	1.8 (0.3)	

Results are presented as mean and standard deviation (SD) for continuous variables with normal distribution and median and interquartile range (IQR) for continuous variables without normal distribution.

BMI, body mass index; DDI, daily dose of insulin; HbA1c, glycated hemoglobin; ACR, albumin/creatinine ratio; AST, aspartate aminotransferase; ALT, alanine aminotransferase; GGTP, gamma-glutamyltransferase; NT-proBNP, N-terminal prohormone of brain natriuretic peptide; CRP, C-reactive protein; eGFR, estimated glomerular filtration rate; LDL, low-density lipoprotein; HDL, high-density lipoprotein; non-HDL, non-high-density lipoprotein; TG, triglycerides; TSH, thyroid-stimulating hormone; LAP, index lipid accumulation product; FLI, fatty liver index; GDF-15, growth differentiation factor 15; APOC3, apolipoprotein C3; RVDD, right ventricular diameters at diastole; IVSD, intraventricular septum thickness at end-diastole; LVDD, left ventricular diameters at diastole; LVSD, left ventricular diameters at systole; EF, ejection fraction; LA vol, left atrium volume; LA vol, index left atrium volume index; mitral E, early diastolic mitral inflow velocity; mitral A, late diastolic mitral inflow velocity; mitral DT, deceleration time of mitral E velocity; LV EDV, left ventricle end-diastolic volume; LV ESV, left ventricle end-systolic volume; LV mass, left ventricle mass; LV mass index, left ventricle mass index; TDI bas sept e′, septal mitral annulus early diastolic velocity; TDI bas sept s′, septal mitral annulus peak systolic velocity; TDI bas sept a′, septal mitral annulus peak late diastolic velocity; TDI bas lat e′, lateral mitral annulus early diastolic velocity; TDI lat sept s′, lateral mitral annulus peak systolic velocity; TDI bas lat a′, lateral mitral annulus peak late diastolic velocity; RV TAM, tricuspid annular motion; Vp flow, velocity propagation at mitral annulus; LVLAVR, left ventricular (LV)–left atrial (LA) volume ratio.

On average, the study participants did not meet the requirements for optimal diabetes control, as their median glycated hemoglobin (HbA1c) was 8.05%. The patients on median used 40 U of insulin daily (IQR 32–56 U), and the median daily insulin dose per kilogram of body weight was 0.62 U/kg (IQR 0.46–0.73 U/kg).

### 3.1 Insulin resistance

The insulin-resistant study participants did not differ from the non-insulin-resistant group in terms of age, years of diabetes duration, HbA1c level, and renal and hepatic performance as well as concentrations of NT-proBNP, GDF-15, or APOC3 (**Table 2**). The participants in the IR group had a higher BMI (29.3 vs. 22.9 kg/m^2^, *p* = 0.000) than the non-IR patients, and their waist circumference indicated abdominal obesity (110 vs. 78.5 cm, *p* = 0.000). Furthermore, they presented with a worsened lipid profile, namely, elevated levels of total cholesterol (5.51 vs. 4.71 mmol/L, *p* = 0.015), triglycerides (TG; 149.7 vs. 76.2 mg/dL, *p* = 0.001), low-density lipoproteins (LDL; 3.46 vs. 2.76 mmol/L, *p* = 0.004), and nonhigh-density lipoproteins (non-HDL 4.06 vs. 3.17 mmol/L, *p* = 0.003). Moreover, subjects with IR had significantly higher inflammation marker levels (CRP 4.3 vs. 1.35 mg/L, *p* = 0.017).

**Table 2:** Comparison of groups with LAP ≥42.5 and LAP <42.5.

Parameter	LAP <42.5 (*n* = 44)	LAP ≥42.5 (*n* = 11)	*p* value
Baseline characteristics			
Age, years (mean, SD)	37.66 (9.72)	39.18 (9.20)	0.64082
Height, cm (mean, SD)	167 (9)	176 (12)	**0.00869**
Body weight, kg (median, IQR)	65.00 (58.00–71.50)	94.00 (80.00–105.00)	**0.00002**
Waist circumference, cm (median, IQR)	78.50 (73.50–85.00)	110.00 (92.00–118.00)	**0.00001**
BMI, kg/m^2^ (median, IQR)	22.98 (21.47–24.86)	29.30 (27.04–32.41)	**0.00026**
Diabetes duration, years (mean, SD)	22.3 (12.5)	19.5 (4.3)	0.45543
DDI, U (median, IQR)	35.50 (31.00–49.00)	58.00 (48.00–73.00)	**0.00143**
DDI per body weight kg, U/kg (median, IQR)	0.58 (0.46–0.74)	0.64 (0.48–0.73)	0.41187
Biochemistry			
HbA1c, % (median, IQR)	8.15 (7.20–10.00)	7.90 (6.70–9.90)	0.74432
ACR, mg/g (median, IQR)	5.00 (2.65–14.85)	3.80 (2.50–7.70)	0.59158
AST, U/L (median, IQR)	19.75 (16.45–25.70)	22.70 (17.10–36.70)	0.23450
ALT, U/L (median, IQR)	16.75 (12.75–25.25	21.60 (18.70–35.00)	0.10295
GGTP, U/L (median, IQR)	17.35 (11.40–30.45)	36.20 (15.80–45.50)	0.05967
NT-proBNP, pg/mL (median, IQR)	43.75 (23.95–105.30)	46.70 (28.50–52.90)	0.85805
CRP, mg/L (median, IQR)	1.35 (0.70–3.45)	4.30 (1.50–7.90)	**0.01693**
Uric acid, umol/L (median, IQR)	248.65 (200.00–323.40)	288.10 (217.90–328.00)	0.37118
eGFR, mL/min/1.73 m^2^ (mean, SD)	105.4 (20.6)	102.6 (17.9)	0.67977
Total cholesterol, mmol/L (mean, SD)	4.71 (0.88)	5.51 (1.16)	**0.01464**
LDL, mmol/L (mean, SD)	2.76 (0.63)	3.46 (0.89)	**0.00366**
HDL, mmol/L (mean, SD)	1.55 (0.36)	1.45 (0.25)	0.41602
Non-HDL, mmol/L (mean, SD)	3.17 (0.78)	4.06 (1.16)	**0.00328**
TG, mg/dL (median, IQR)	76.17 (59.34–112.48)	149.68 (110.71–191.31)	**0.00056**
TSH, µIU/mL (median, IQR)	1.61 (0.93–2.71)	1.18 (0.78–1.65)	0.29071
Insulin resistance			
LAP index (median, IQR)	16.59 (10.10–27.41)	79.92 (57.20–93.08)	**0.00000**
Experimental biomarkers			
GDF-15, pg/mL (median, IQR)	284.91 (109.42–551.15)	452.47 (267.86–512.99)	0.28797
APOC3, ng/mL (median, IQR)	22.19 (13.73–34.79)	23.49 (17.09–31.16)	0.77637
Echocardiography			
RVDD, mm (mean, SD)	25 (3)	27 (2)	**0.01397**
IVSD, mm (mean, SD)	13 (2)	14 (1)	0.06223
LVDD, mm (mean, SD)	45 (4)	51 (3)	**0.00006**
LVSD, mm	29 (3)	33 (3)	**0.00181**
EF, % (mean, SD)	65% (5%)	64% (4%)	0.54689
LA vol, mL (mean, SD)	41 (9)	56 (11)	**0.00007**
LA vol index, mL/m^2^ (mean, SD)	23 (4)	26 (5)	0.10593
Mitral E, cm/s (mean, SD)	84 (19)	72 (20)	0.10262
Mitral A, cm/s (mean, SD)	61 (15)	73 (19)	0.05375
Mitral E/A (median, IQR)	1.298 (1.108–1.704)	0.944 (0.877–1.216)	**0.00330**
Mitral DT, ms (median, IQR)	232 (204.5–245)	220 (185–270)	0.55477
LV EDV, mL (mean, SD)	75 (15)	87 (11)	**0.02540**
LV ESV, mL (mean, SD)	27 (6)	32 (6)	**0.03222**
LV mass, g (mean, SD)	119 (30)	169 (24)	**0.00003**
LV mass index, g/m^2^ (mean, SD)	68 (14)	79 (6)	**0.03061**
TDI bas sept e′, cm/s (mean, SD)	12 (3)	9 (2)	**0.00792**
TDI bas sept s′, cm/s (median, IQR)	8 (8–9)	9 (8–9)	0.63795
TDI bas sept a′, cm/s (median, IQR)	8 (7–9)	9 (8–10)	**0.02467**
TDI bas lat e′, cm/s (median, IQR)	14 (13–15)	10 (9–16)	0.10114
TDI bas lat s′, cm/s (median, IQR)	10 (9–13)	8 (7–12)	**0.01863**
TDI bas lat a′, cm/s (mean, SD)	8 (2)	9 (3)	0.23141
Mean e′, cm/s (mean, SD)	13 (3)	11 (4)	0.11550
E/e′ (median, IQR)	6.8 (5.0–8.0)	6.9 (6.0–8.0)	0.67082
RV TAM, mm (mean, SD)	26 (3)	27 (2)	0.42854
Vp, cm/s (mean, SD)	50 (6)	51 (8)	0.56735
LVLAVR (mean, SD)	1.86 (0.32)	1.61 (0.36)	**0.04143**

Results are presented as mean and standard deviation (SD) for continuous variables with normal distribution and median and interquartile range (IQR) for continuous variables without normal distribution. Values in bold type are statistically significant at p <0.05.

BMI, body mass index; DDI, daily dose of insulin; HbA1c, glycated hemoglobin; ACR, albumin/creatinine ratio; AST, aspartate aminotransferase; ALT, alanine aminotransferase; GGTP, gamma-glutamyltransferase; NT-proBNP, N-terminal prohormone of brain natriuretic peptide; CRP, C-reactive protein; eGFR, estimated glomerular filtration rate; LDL, low-density lipoprotein; HDL, high-density lipoprotein; non-HDL, non-high-density lipoprotein; TG, triglycerides; TSH, thyroid-stimulating hormone; LAP index, lipid accumulation product; FLI, fatty liver index; GDF-15, growth differentiation factor 15; APOC3, apolipoprotein C3; RVDD, right ventricular diameters at diastole; IVSD, intraventricular septum thickness at end-diastole; LVDD, left ventricular diameters at diastole; LVSD, left ventricular diameters at systole; EF, ejection fraction; LA vol, left atrium volume; LA vol index, left atrium volume index; mitral E, early diastolic mitral inflow velocity; mitral A, late diastolic mitral inflow velocity; mitral DT, deceleration time of mitral E velocity; LV EDV, left ventricle end-diastolic volume; LV ESV, left ventricle end-systolic volume; LV mass, left ventricle mass; LV mass index, left ventricle mass index; TDI bas sept e′, septal mitral annulus early diastolic velocity; TDI bas sept s′, septal mitral annulus peak systolic velocity; TDI bas sept a′, septal mitral annulus peak late diastolic velocity; TDI bas lat e′, lateral mitral annulus early diastolic velocity; TDI lat sept s′, lateral mitral annulus peak systolic velocity; TDI bas lat a′, lateral mitral annulus peak late diastolic velocity; RV TAM, tricuspid annular motion; Vp, flow velocity propagation at mitral annulus; LVLAVR, left ventricular (LV)–left atrial (LA) volume ratio.

### 3.2 Echocardiography

In echocardiograms, the IR patients presented with significantly higher LV mass index (79 vs. 68 g/m^2^, *p* = 0.030), left ventricle volumes (left ventricle end-diastolic volume 87 vs. 75 mL, *p* = 0.025), and left ventricle end-systolic volume (32 vs. 27 mL, *p* = 0.032). The IR and non-IR study participants did not differ in terms of indexed left atrium volume, early diastolic mitral inflow velocity (mitral E), late diastolic mitral inflow velocity (mitral A), or median medial mitral annulus early diastolic velocity (e′). However, the IR patients had a significantly lower mitral E/A ratio (0.944 vs. 1.298, *p* = 0.003), a possible indication of worsened diastolic function. Patients with LAP exceeding 42.5 exhibited a pattern of significantly reduced peak systolic mitral annular velocity at the lateral part of the mitral annulus (TDI bas lat s′; 8 vs. 10 cm/s, *p* = 0.019) and peak early diastolic mitral annular velocity at the septal part of the mitral annulus (TDI bas sept e′; 9 vs. 12 cm/s, *p* = 0.008) with increased peak late diastolic mitral annular velocity at the septal part of the mitral annulus (TDI bas sept a′; 9 vs. 8 cm/s, *p* = 0.0246). In the IR group, the left ventricular (LV)–left atrial (LA) volume ratio (LVLAVR) at the end of diastole was significantly lower (1.61 vs. 1.86, *p* = 0.041).

A further analysis using Spearman’s r correlation (**Table 3**) revealed the different patterns of parameters correlating with mitral E/A ratio and mitral E/e′ ratio. The mitral E/A ratio correlated moderately with LAP index (*r* = -0.4112, *p* = 0.0068) and weakly with TG concentration and waist circumference (*r* = -0.3392, *p* = 0.0215, *r* = -0.3581, *p* = 0.0215, respectively). At the same time, mitral E/e′ correlated moderately with age (*r* = 0.5544, *p* = 0.0001) as well as weakly correlated with APOC3 (*r* = 0.3099, *p* = 0.0458) and NT-proBNP (*r* = 0.3713, *p* = 0.0155).

**Table 3:** Spearman’s *R* correlations for mitral E/A ratio and mitral E/e′ ratio.

	Mitral E/A ratio		Mitral E/e′ ratio	
	Spearman’s *R*	*p*	Spearman’s *R*	*p*
Age	-0.2976	0.0556	0.5544	0.000169
GDF-15	-0.0233	0.8837	0.0360	0.8229
APOC3	0.0293	0.2323	0.3099	0.0458
eGFR	-0.0864	0.5818	-0.0593	0.7203
Urea	-0.0220	0.8883	-0.1079	0.4966
AST	-0.0874	0.5772	-0.0121	0.9394
ALT	-0.2376	0.1250	-0.0787	0.6205
GGTP	-0.3071	0.0452	-0.0046	0.9769
NT-proBNP	-0.0261	0.8683	0.3713	0.0155
Uric acid	-0.1446	0.8683	-0.0105	0.9475
ACR	-0.1156	0.4606	-0.0064	0.9676
HbA1c	0.0279	0.8796	-0.0938	0.5542
CRP	-0.1958	0.8796	0.1609	0.3088
LAP index	-0.4112	0.0068	0.1036	0.5193
TSH	0.0924	0.5919	-0.0146	0.9337
Total cholesterol	0.0364	0.8168	-0.1752	0.2669
LDL	-0.0322	0.8397	-0.6977	0.4895
HDL	-0.0754	0.6306	-0.1860	0.4895
Non-HDL	0.0148	0.9249	-0.1153	0.2382
TG	-0.3392	0.0261	0.0008	0.9959
BMI	-0.2803	0.0722	0.3368	0.0313
Waist circumference	-0.3581	0.0215	0.1457	0.3634
Diabetes duration	-0.0426	0.7889	0.3024	0.0546
DDI/kg	-0.0644	0.6853	0.0565	0.7255

### 3.3 Contribution of anthropometric and laboratory parameters to diastolic heart function

We did not find significant differences between the median BMI and insulin resistance among the study participants divided into subgroups with normal weight, overweight, and obese. Comparing the BMI categories, insulin resistance seems to correlate with the mitral E/A ratio only in the overweight group (**Tables 4**, **5**). In univariable linear regression analysis, IR significantly correlated with mitral E/A with corrected *R* ^2 =^ 0.2835 and *p* = 0.0432.

**Table 4:** Comparison between the BMI in subgroups by BMI category and LAP index.

Parameter	LAP <42.5	LAP ≥42.5	*p* value
BMI <25 kg/m^2^	21.96 (21.16–23.35)	18.92 ^*^	^*^
BMI 25–30 kg/m2	26.98 (25.99–28.15)	28.51 (27.04–29.01)	0.2134
BMI > 30 kg/m2	32.46 (30.08–34.84)	32.41 (31.35–34.72)	0.8464

^*^Only one participant with BMI < 25 kg/m^2^ had LAP ≥42.5.

**Table 5:** Univariable regression models for mitral E/A ratio according to BMI category.

Mitral E/A ratio						
	**BMI < 25 kg/m^2^** Corrected R^2^ = -0.0388 *p-*value = 0.7522		**BMI 25 – 30 kg/m^2^** Corrected R^2^ = 0.2835 *p-*value = 0.0432		**BMI > 30 kg/m^2^** Corrected R^2^ = 0.4811 *p-*value = 0.1185	
**LAP index**	*t* value	*p*	*t* value	*p*	*t* value	*p*
	-0.03887	0.7522	-2.3137	0.0432	-2.1700	0.1185

**Table 6** depicts univariable and multivariable regression models for the mitral E/A ratio and the LVLAVR. In univariable linear regression models, age, LAP index, and FLI score were negatively associated with the mitral E/A ratio. No other univariable models reached a statistically significant threshold of *p <*0.05. *A* multivariable regression model including LAP index, age, ALT, uric acid, HbA1c, CRP, total cholesterol, LDL, BMI, and diabetes duration explained almost 62% of mitral E/A variation (adjusted *R* ^2^ for the model: 0.619, *p* = 0.000). Notably, LAP index, age, and ALT, uric acid, CRP, and LDL concentrations negatively correlated with mitral E/A ratio. Additionally, HbA1c, diabetes duration, BMI, and total cholesterol level correlated positively with the E/A ratio.

**Table 6:** Univariable and multivariable linear regression models for mitral E/A ratio and LVLAVR.

	Mitral E/A ratio				LVLAVR			
	Univariable analysis		Multivariable analysis Corrected *R* ^2^ 0.618711 *p* = 0.000084		Univariable analysis		Multivariable analysis Corrected *R* ^2^ 0.478627 *p* = 0.000941	
	*t* value	*p*	*t* value	*p*	*t* value	*p*	*t* value	*p*
Age	-2.3183	0.0256	-4.7846	0.0000	-2.6597	0.0113	-2.5554	0.0170
Sex (female)	0.6831	0.4985			-1.5985	0.1182		
GDF-15	-0.1572	0.8758			-0.9478	0.3492		
APOC3	0.3116	0.7569			-1.2085	0.2341		
eGFR	-0.6702	0.5065			-0.0674	0.9465	-2.7752	0.0102
Urea	-0.6333	0.53			0.5078	0.6144		
AST	0.5903	0.5583			0.5903	0.5583		
ALT	-1.0942	0.2803	-3.3088	0.0029	0.54799	0.5868		
GGTP	-0.543	0.59			-0.7846	0.4374		
NT-proBNP	0.204	0.8393			0.2101	0.8346		
Uric acid	-1.0553	0.2974	-2.7264	0.0117	0.1865	0.8529		
ACR	-0.5565	0.5809			1.3236	0.1933		
HbA1c	0.9709	0.3373	3.7629	0.0009	1.118	0.2704		
CRP	-1.1644	0.2509	-3.0705	0.0052	1.4928	0.1435	2.6038	0.0152
LAP index	-2.7227	0.0095	-3.2982	0.0030	-1.8771	0.0681	-3.1956	0.0037
TSH	0.249	0.8048			-0.4283	0.6712		
Total cholesterol	0.1764	0.8608	4.5692	0.0001	0.0896	0.9290	-3.1898	0.0038
LDL	-0.2505	0.8035	-4.7846	0.0004	0.4547	0.6518	3.1536	0.0041
HDL	0.4362	0.6649			-0.0485	0.9615		
Non-HDL	0.0352	0.972			0.1167	0.9076		
TG	-1.0828	0.2852			1.0795	0.2869	-2.1369	0.0425
FLI					-1.8093	0.0783		
BMI	-2.0181	0.0503	2.9932	0.0063	-2.7126	0.0099		
Waist circumference					-3.03	0.0043		
Diabetes duration	-0.6377	0.5272	2.3620	0.0266	-0.7237	0.4736		
DDI/kg	0.1633	0.8711			1.7575	0.0868		

In the univariable analysis, we observed a negative association of age, BMI, and waist circumference with LVLAVR. In the multivariable regression model, combined LAP index and age, together with eGFR, CRP, total cholesterol, LDL, and TG concentrations, explained nearly 48% of LVLAVR variation (adjusted *R* ^2 =^ 0.479, *p* = 0.001).

## 4 Discussion

The present study stems from the growing number of observations considering the rising rates of so-called double diabetes, which describes patients with T1D presenting with features of insulin resistance traditionally linked to type 2 diabetes (T2D) (9–11). There seems to be a two-directional correlation between insulin resistance and heart dysfunction. As already described, insulin resistance may predict HF development. Nevertheless, heart failure presence may also precede the occurrence of insulin resistance and increase the risk of T2D (22). This study does not provide sufficient information about the timing of the occurrence of these particular disorders. Nevertheless, based on the magnitude of changes and the relatively young age of the study participants, we hypothesize that insulin resistance may contribute more to heart dysfunction than heart dysfunction to insulin resistance in this particular case. However, further longitudinal studies on larger groups are required to address the specific timeline of the interconnections between insulin resistance and heart dysfunction in patients with T1D.

This study provides evidence of worsened mitral E/A ratio, subclinical changes in left ventricular function (e′, a′, and s′), and significant lowering of the LVLAVR among patients with type 1 diabetes and IR. Approximately up to 67% of T1D patients suffer from asymptomatic HF, and up to 15% suffer from symptomatic heart failure (18, 19). Among patients with T1D, diabetes seems to accelerate the development of HF, which was described in a recent study in which the prevalence of symptomatic HF in patients with T1D was comparable to the general population older by 10–23 years (3, 4). It is worth mentioning that the participants in our study categorized by age seemed to present with median mitral E/A ratios of patients at least a decade older than they actually were (23–25). An expanding body of evidence proves that early cardiac myopathy indicators may be present even in the pediatric population in spite of tight metabolic control (5).

Observations from our study concerning cardiac performance among patients with T1D stay in line with patterns that have already described (26, 27). However, our findings add a new perspective to this well-established problem. As previously shown in other publications, patients presenting with insulin resistance are at a higher risk of diastolic heart dysfunction compared to non-insulin-resistant ones (28). In our study, patients with T1D and IR recognized as LAP ≥42.5 had a significantly lower mitral E/A ratio compared to non-insulin-resistant participants. The study group with IR did not differ from the non-IR group in terms of age, diabetes duration, glycemic control, or daily dose of insulin per kilogram of body weight. Moreover, the correlation between insulin resistance and mitral E/A ratio was evident only in the overweight group. Nevertheless, this novel notion is seemingly not explained yet.

In this study, Spearman’s *R* correlations for mitral E/A and mitral E/e′ ratios revealed a compelling aspect of heart remodeling in type 1 diabetes. Among study participants, the mitral E/A ratio correlated moderately with the LAP index, while the mitral E/e′ did not correlate with insulin resistance surrogate. It significantly correlated instead with age, APOC3, and NT-proBNP. Due to its design, data from this study do not allow us to draw further conclusions. Nevertheless, we consider this finding worthy of further exploration.

Multivariable regression for the mitral E/A ratio in our study seems to be a model integrating multiple hypotheses on the cardiological consequences of metabolic disturbances associated with diabetes and insulin resistance. The main finding of this model is that an insulin resistance surrogate, the LAP index, correlates with the mitral E/A ratio in patients with T1D. Secondly, the mitral E/A ratio in patients with T1D may be possibly associated with traditional predictors of worsened heart function already mentioned in the literature, such as age, HbA1c, total cholesterol and LDL, BMI, and diabetes (25). Thirdly, our model presents the correlation between the uric acid concentrations and mitral E/A ratio. Previously published data from other studies links hyperuricemia to an increase in both inflammation and oxidative stress and therefore fuel cardiac remodeling and progression into diastolic dysfunction (29). Data from our study seems to affirm this notion.

In our study, patients with IR had significantly decreased s′ and e′ and significantly increased a′ in tissue Doppler imaging (TDI), which suggests cardiac stiffening early in the course of heart dysfunction (30). We hypothesize that insulin resistance accelerates the pattern of typical changes previously described in both animal models of diabetic cardiomyopathy and studies on patients with T1D (3, 29).

Given that LVLAVR seems to correlate with major adverse cardiac events (MACE) and reflect age-related heart remodeling, we decided to incorporate it into our study (31, 32). Although the data on LVLAVR values corresponding with an increased risk of MACE is conflicting, the LVLAVR values less than 2.5–3.3 reportedly correlate with an increased risk of MACE, and this effect grows as the LVLAVR decreases (17, 31, 33, 34). In our study group, the mean LVLAVR was 1.8 and was significantly lower in the IR group compared to the non-IR group (1.61 vs. 1.86, *p* = 0.0414), which, we hypothesize, may be a surrogate of an increased MACE risk in IR patients with T1D. In multivariable regression analysis LAP index, age, eGFR, and concentrations of total cholesterol, LDL, and TG were negatively associated with LVLAVR. Surprisingly, CRP and LDL cholesterol levels correlated positively with LVLAVR. Although LVLAVR use in diabetic patients has not been validated yet, we consider these findings to be worthy of further exploration.

Additionally, in our study cohort, we observed significantly higher levels of CRP among IR patients. This finding supports the possible link between systemic low-grade inflammation and insulin resistance and a potential relatedness between CRP overexpression and worsened diastolic heart function (35).

Nevertheless, there are limitations to this study. First of all, this was a relatively small single-center study. Therefore, further multicenter studies on larger cohorts are needed to gather more comprehensive data. Second, as the patients using any medications apart from insulin made up less than 25% of the group, we could not sufficiently incorporate these data in statistical analyses. As a consequence, we decided to retract it from the study. Larger-sample studies are needed to describe the influence of medication use on cardiac parameters among young patients with T1D. Third, due to organizational challenges, we decided to use a surrogate index of insulin resistance instead of the gold standard hyperinsulinemic–euglycemic clamp. However, lipid accumulation product (LAP) has been previously validated against the gold standard and offers satisfactory sensitivity and specificity. Finally, longitudinal studies are necessary to explore the impact of the parameters and models described in this study on the risk of cardiac events.

Insulin resistance seems to accelerate the pattern of typical changes in heart function among patients with type 1 diabetes. Compared to non-insulin-resistant participants, patients with type 1 diabetes and insulin resistance present with significantly worsened mitral E/A ratio and distinctly affected mitral annulus velocities. Insulin resistance correlates with mitral E/A ratio in an overweight subgroup, but seemingly not in normal-weight and obese participants. This finding is not yet explained. The left ventricular and left atrial volume ratio (LVLAVR), novel yet not validated in the diabetic population index, seems to hold a premise to stratify patients into subgroups at a specific risk of adverse cardiac outcomes. However, further analyses are needed to confirm this premise.

## Data availability statement

The raw data supporting the conclusions of this article will be made available by the authors, without undue reservation.

## Ethics statement

The studies involving humans were approved by Bioethics Committee of the Medical University of Lodz. The studies were conducted in accordance with the local legislation and institutional requirements. The participants provided their written informed consent to participate in this study.

## Author contributions

KCz: Conceptualization, Data curation, Formal analysis, Investigation, Methodology, Project administration, Resources, Validation, Writing – original draft, Writing – review & editing. ZS: Data curation, Formal analysis, Investigation, Methodology, Resources, Writing – original draft, Writing – review & editing. AB: Conceptualization, Data curation, Formal analysis, Investigation, Methodology, Resources, Validation, Writing – original draft, Writing – review & editing. JD: Conceptualization, Data curation, Formal analysis, Investigation, Methodology, Resources, Writing – original draft, Writing – review & editing. KCy: Conceptualization, Data curation, Formal analysis, Investigation, Methodology, Project administration, Resources, Supervision, Validation, Writing – original draft, Writing – review & editing.
